# Mexiletine-like cellular electrophysiological effects of GS967 in canine ventricular myocardium

**DOI:** 10.1038/s41598-021-88903-3

**Published:** 2021-05-05

**Authors:** Tamás Hézső, Muhammad Naveed, Csaba Dienes, Dénes Kiss, János Prorok, Tamás Árpádffy-Lovas, Richárd Varga, Erika Fujii, Tanju Mercan, Leila Topal, Kornél Kistamás, Norbert Szentandrássy, János Almássy, Norbert Jost, János Magyar, Tamás Bányász, István Baczkó, András Varró, Péter P. Nánási, László Virág, Balázs Horváth

**Affiliations:** 1 Department of Physiology, Faculty of Medicine, University of Debrecen, Nagyerdei krt 98, 4012 Debrecen, Hungary; 2 Department of Pharmacology and Pharmacotherapy, Faculty of Medicine, University of Szeged, Dóm tér 12, 6701 Szeged, Hungary; 3 MTA-SZTE Research Group for Cardiovascular Pharmacology, Hungarian Academy of Sciences, Szeged, Hungary; 4 Department of Biophysics, School of Medicine, Akdeniz University, Antalya, Turkey; 5 Department of Basic Medical Sciences, Faculty of Dentistry, University of Debrecen, Debrecen, Hungary; 6 Division of Sport Physiology, Department of Physiology, Faculty of Medicine, University of Debrecen, Debrecen, Hungary; 7 Department of Pharmacology and Pharmacotherapy, Interdisciplinary Excellence Centre, University of Szeged, Szeged, Hungary; 8 Department of Dental Physiology and Pharmacology, Faculty of Dentistry, University of Debrecen, Debrecen, Hungary; 9 Faculty of Pharmacy, University of Debrecen, Debrecen, Hungary

**Keywords:** Drug discovery, Physiology, Cardiology

## Abstract

Enhancement of the late Na^+^ current (I_NaL_) increases arrhythmia propensity in the heart, while suppression of the current is antiarrhythmic. GS967 is an agent considered as a selective blocker of I_NaL_. In the present study, effects of GS967 on I_NaL_ and action potential (AP) morphology were studied in canine ventricular myocytes by using conventional voltage clamp, action potential voltage clamp and sharp microelectrode techniques. The effects of GS967 (1 µM) were compared to those of the class I/B antiarrhythmic compound mexiletine (40 µM). Under conventional voltage clamp conditions, I_NaL_ was significantly suppressed by GS967 and mexiletine, causing 80.4 ± 2.2% and 59.1 ± 1.8% reduction of the densities of I_NaL_ measured at 50 ms of depolarization, and 79.0 ± 3.1% and 63.3 ± 2.7% reduction of the corresponding current integrals, respectively. Both drugs shifted the voltage dependence of the steady-state inactivation curve of I_NaL_ towards negative potentials. GS967 and mexiletine dissected inward I_NaL_ profiles under AP voltage clamp conditions having densities, measured at 50% of AP duration (APD), of −0.37 ± 0.07 and −0.28 ± 0.03 A/F, and current integrals of −56.7 ± 9.1 and −46.6 ± 5.5 mC/F, respectively. Drug effects on peak Na^+^ current (I_NaP_) were assessed by recording the maximum velocity of AP upstroke (V^+^_max_) in multicellular preparations. The offset time constant was threefold faster for GS967 than mexiletine (110 ms *versus* 289 ms), while the onset of the rate-dependent block was slower in the case of GS967. Effects on beat-to-beat variability of APD was studied in isolated myocytes. Beat-to-beat variability was significantly decreased by both GS967 and mexiletine (reduction of 42.1 ± 6.5% and 24.6 ± 12.8%, respectively) while their shortening effect on APD was comparable. It is concluded that the electrophysiological effects of GS967 are similar to those of mexiletine, but with somewhat faster offset kinetics of V^+^_max_ block. However, since GS967 depressed V^+^_max_ and I_NaL_ at the same concentration, the current view that GS967 represents a new class of drugs that selectively block I_NaL_ has to be questioned and it is suggested that GS967 should be classified as a class I/B antiarrhythmic agent.

## Introduction

Following the large Na^+^ current surge associated with the AP upstroke (called I_NaP_) a smaller but sustained current component (called I_NaL_) remains active throughout the entire cardiac AP. Enhanced I_NaL_, due to diseases like heart failure^1–3^, hypertrophic cardiomyopathy^4^ or LQT3^5^, is known to increase arrhythmia propensity in the heart^1,6–8^. It has been known for a long time that many of the class I antiarrhythmic drugs inhibit I_NaL_ in addition to blocking I_NaP_^9–14^. This latter effect, however, was considered to enhance proarrhythmic risk and consequently the incidence of sudden cardiac death^15^. As a consequence, development of agents to suppress I_NaL_ selectively was a straightforward strategy in the past decades^2,16–19^. One of these agents, 6-(4-(trifluoromethoxy)phenyl)-3-(trifluoromethyl)-[l,2,4]triazolo[4,3-a]pyridine, known as GS967 or GS458967, was reported to be a particularly selective candidate when tested in rabbit ventricular cells^16^. It was also shown that important species-dependent variations existed in the electrophysiological properties of myocardial preparations^20,21^, and canine ventricular myocytes are considered a reasonably good model for human ventricular cells^22–24^. Furthermore, it was previously shown using the action potential voltage clamp technique that the kinetic properties of I_NaL_ are similar in dogs and humans^20^, while differing from those of other mammals including guinea pigs, rabbits and pigs^25–27^. Therefore, we studied the effects of GS967 on I_NaL_ and AP V^+^_max_ (the indicator of I_NaP_), comparing them to similar effects of the class I/B antiarrhythmic agent mexiletine in canine ventricular preparations. In this work, based on experimental evidence, we challenge the present concept that GS967 exerts a selective I_NaL_ blocking effect, since it has very similar class I/B antiarrhythmic properties as mexiletine.

## Methods

### Animals

Adult mongrel dogs of either sex were anesthetized with i.m. injections of 10 mg/kg ketamine hydrochloride (Calypsol, Richter Gedeon, Hungary) + 1 mg/kg xylazine hydrochloride (Sedaxylan, Eurovet Animal Health BV, The Netherlands) according to a protocol approved by the local Animal Care Committees (license N^o^: 9/2015/DEMÁB at University of Debrecen; and I-74-15-2017, I-74-24-2017 at University of Szeged) and by the Department of Animal Health and Food Control of the Ministry of Agriculture and Rural Development (XIII/3330/2017 and XIII/3331/2017). All animal procedures conform to the guidelines from Directive 2010/63/EU of the European Parliament on the protection of animals used for scientific purposes and the Guide for the Care and Use of Laboratory Animals (USA NIH publication NO 85-23, revised 1996) The study was carried out in compliance with the ARRIVE guidelines.

### Isolation of cardiomyocytes

Single canine myocytes were obtained by enzymatic dispersion using the segment perfusion technique, as previously described^28^. Briefly, a wedge-shaped section of the ventricular wall supplied by the LAD coronary artery was cannulated, dissected and perfused with a nominally Ca^2+^-free Joklik solution (Minimum Essential Medium Eagle, Joklik Modification, Sigma-Aldrich Co. St. Louis, MO, USA) for a period of 5 min. After this, the tissue was perfused with Joklik solution supplemented with 1 mg/ml collagenase (Type II, Worthington Biochemical Co., Lakewood, NJ, USA; representing final activity of 224 U/ml) and 0.2% bovine serum albumin (Fraction V., Sigma) containing 50 µM Ca^2+^ for 30 min. Finally, the normal external Ca^2+^ concentration was gradually restored and the cells were stored at 15 °C in Minimum Essential Medium Eagle until use. The chemicals used in the experiments were obtained from Sigma-Aldrich Co. (St. Louis, MO, USA), except for GS967, which was purchased from MedKoo Biosciences, Inc. (Morrisville, NC, USA).

### Electrophysiology

Cells were placed in a plexiglass chamber under an inverted microscope, allowing for continuous superfusion with a modified Tyrode solution by gravity flow at a rate of 1–2 ml/min. The modified Tyrode solution contained (in mM): NaCl 121, KCl 4, CaCl_2_ 1.3, MgCl_2_ 1, HEPES 10, NaHCO_3_ 25, glucose 10 at pH = 7.35, which was supplemented according to the actual experimental design. The osmolarity of this solution was 300 ± 3 mOsm, measured with a vapor pressure osmometer. In all experiments, the bath temperature was set to 37 ºC using a temperature controller (Cell MicroControls, Norfolk, VA, USA). Electrical signals were amplified and recorded (MultiClamp 700A or 700B, Molecular Devices, Sunnyvale, CA, USA) under the control of a pClamp 10 software (Molecular Devices) following analogue–digital conversion (Digidata 1440A or 1332, Molecular Devices). Electrodes, having tip resistances of 2–3 MΩ when filled with pipette solution, were manufactured from borosilicate glass. Transmembrane currents were recorded in whole-cell voltage clamp configuration. The series resistance was typically 4–8 MΩ, and the measurement was discarded if it changed substantially during the experiment.

#### Action potential voltage clamp

Action potential voltage clamp experiments were performed according to the methods described^29,30^. A previously recorded midmyocardial canine ventricular AP was applied as command signal and the current traces were recorded continuously in modified Tyrode solution before and after 5 min superfusion with the Na^+^ channel inhibitor applied. The drug-sensitive current was obtained by subtracting the post-drug trace from the reference pre-drug trace. When measuring the effects of GS967 or mexiletine on I_NaL_, the experiments were performed in the presence of 1 µM nisoldipine + 1 µM E4031 + 100 µM chromanol 293B, added to the Tyrode solution, in order to eliminate any interference from Ca^2+^ and K^+^ currents. The pipette solution contained (in mM): K-aspartate 120, KCl 30, MgATP 3, HEPES 10, Na_2_-phosphocreatine 3, EGTA 0.01, cAMP 0.002, KOH 10 at pH = 7.3 with an osmolarity of 285 mOsm. The amplitude of the dissected I_NaL_ was evaluated at 50% duration of the APD_90_ value of the command AP. When determining the current integral, the initial 20 ms after the AP upstroke was excluded from evaluation in order to eliminate the contribution of I_NaP_. In each experiment 20 consecutive current traces were averaged and analyzed in order to reduce the noise and the trace-to-trace fluctuations of action potential configuration. Ion currents were normalized to cell capacitance, determined in each cell by applying hyperpolarizations from +10 to −10 mV for 15 ms.

#### Conventional voltage clamp

Conventional voltage clamp experiments, using rectangular command pulses, were performed to study the effects of GS967 and mexiletine on I_NaL_ at stable test potentials. The external solution was a HEPES-buffered Tyrode solution containing (in mM): NaCl 144, NaH_2_PO_4_ 0.4, KCl 4.0, CaCl_2_ 1.8, MgSO_4_ 0.53, glucose 5.5 and HEPES 5.0, at pH = 7.4) supplemented with 1 µM nisoldipine, 0.5 µM HMR-1556 and 0.1 µM dofetilide in order to block Ca^2+^ and K^+^ currents. The composition of the pipette solution was (in mM): CsCl 125, TEACl 20, MgATP 5, EGTA 10, HEPES 10, at a pH = 7.2). Test pulses were clamped to −20 mV for 2 s from the holding potential of −120 mV before and after application of GS967 or mexiletine, while the total amount of I_NaL_ was determined by pharmacological subtraction performed by a final superfusion with 20 µM TTX. The amplitude of I_NaL_ was evaluated at 50 ms after beginning the pulse. For determination of current integral the initial 20 ms was excluded from evaluation in order to minimize the contribution of I_NaP_. In order to study the voltage-dependence of steady-state inactivation of I_NaL_, test depolarizations to −20 mV were preceded by a set of 2 s long prepulses clamped to various voltages between −130 and −40 mV for 2 s. Peak currents measured after these prepulses (I_test_) were normalized to the peak current measured after the −130 mV prepulse (I_max_) and these I_test_/I_max_ ratios were plotted against the respective prepulse potentials. The data were fitted to the two-state Boltzmann function (y = 100/[1 + exp((x-V1/2)/k)]).

#### Recording of APs from multicellular preparations

Multicellular preparations (right ventricular papillary muscles) were selected to prevent the limitations of isolated myocyte studies, like the absence of intercellular clefts or potential damage to channel proteins, allowing better representation of in vivo conditions. The experiments were performed as previously described^31^. Briefly, transmembrane potentials were recorded using 3 M KCl filled sharp glass microelectrodes having tip resistance between 10 and 20 MΩ. These electrodes were connected to the input of a high impedance electrometer (MDE GmbH, Heidelberg, Germany). Preparations were paced by a pair of platinum electrodes using 1 ms wide rectangular current pulses with twice the threshold amplitude at 37 °C. The pacing cycle length was set to 1 s for at least 60 min allowing the preparations to equilibrate before starting the experiment.

Following equilibration at 1 s cycle length, the cycle length was sequentially varied between 0.3 and 5 s. At each cycle length the 25th AP was recorded, and the cycle length was then changed. Under these conditions a quasi steady-state rate-dependence could rapidly be obtained. APs were digitized at 100 kHz using an ADA 3300 data acquisition board (Real Time Devices Inc., State Collage, PA, USA) and stored for later analysis. After taking control records at each cycle length the preparations were superfused with either GS967 or mexiletine for 20 min and then the protocol was repeated. Efforts were made to maintain the same impalement throughout each experiment. If, however, an impalement became dislodged, adjustment was attempted, but when the parameters of the re-established impalement deviated by more than 5% from the previous record, the experiment was discarded.

Restitution kinetics of the maximum rate of depolarization (V^+^_max_) is considered as the indicator of the offset time constant. To determine the restitution time constant for V^+^_max_ the preparations were paced using a train of 20 basic stimuli delivered at a basic cycle length of 1 s. Each train was followed by a single extra stimulus applied with successively longer coupling intervals. The train of basic stimuli was reinitiated following the delivery of the extra stimulus. In this way, each 20th basic AP was followed by a single extra AP occurring at gradually increasing diastolic intervals. The diastolic interval was defined as the time from APD_90_ of the last basic member of the train to the upstroke of the extra AP. Recovery curves were generated by plotting the V^+^_max_ of each extra AP as a function of the respective diastolic interval and data were fitted to a single exponential function.

Onset kinetics of drug action on V^+^_max_ were determined by stimulating the preparation at a cycle length of 0.4 s following a few min period of rest and the initial 40 APs were recorded and data were plotted against the number of the analyzed AP within the train. The rate of development of block was obtained by monoexponential fitting of the V^+^_max_ values.

#### Determination of beat-to-beat variability of APD in isolated myocytes

Since beat-to-beat variability of APD is relatively small in multicellular preparations due to the balancing effect of the neighboring cells, these experiments were performed in isolated myocytes. Series of 50 consecutive action potentials were analyzed to estimate the beat-to-beat variability of APD, defined as short term variability (SV), according to the following formula:

$${\text{SV }} = \, \Sigma \, \left( {\left| {{\text{APD}}_{{{\text{i}} + 1}} - {\text{APD}}_{{\text{i}}} } \right|} \right)/ \, \left[ {{\text{n}}_{{{\text{beats}}}} * \, \surd 2} \right]$$

where SV is short term variability, APD_i_ and APD_i+1_ indicate the durations of the ith and i + 1th APs, respectively, at 90% level of repolarization and n_beats_ denotes the number of consecutive beats analyzed^32^. Changes in SV were presented as Poincaré plots where 50 consecutive APD values are plotted, each against the duration of the previous AP.

### Statistics

Results are expressed as mean ± SEM values, n denotes the number of myocytes or multicellular preparations studied. Statistical significance of differences was evaluated using one-way ANOVA followed by Student's t-test for paired or unpaired data as pertinent. Differences were considered significant when p was less than 0.05. Data were processed, analyzed and figures were created in Excel (Microsoft Corp., Redmond, WA, USA), and Origin 2015 (Originlab Corp., Northampton, MA, USA).

## Results

### Effects of GS967 and mexiletine on I_NaL_ using conventional voltage clamp

**Figure 1:**
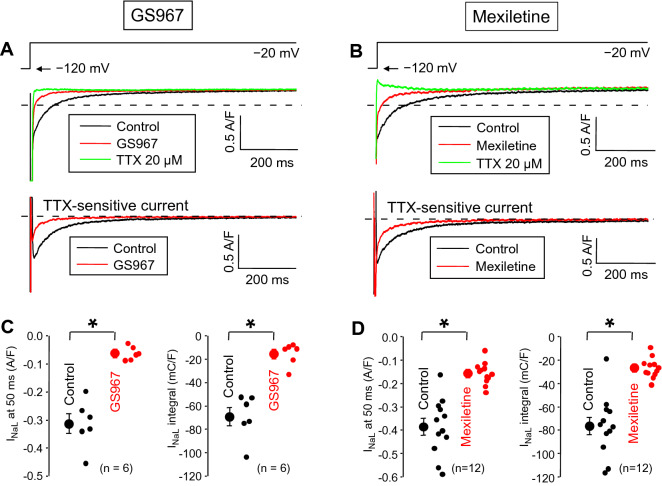
Effects of 1 µM GS967 (**A**,**C**) and 40 µM mexiletine (**B**,**D**) on I_NaL_ under conventional voltage clamp conditions using test pulses of 2 s duration clamped to −20 mV from the holding potential of −120 mV. At the end of each experiment the cells were superfused with 20 µM TTX to dissect the remaining I_NaL_. Dashed lines indicate zero current level. (**A**,**B**) Representative superimposed analogue records. (**C**,**D**) Average I_NaL_ densities (measured at 50 ms) and integrals (measured from 20 ms to 2 s). Symbols and bars are mean ± SEM, small dots represent individual data, numbers in parentheses indicate the number of myocytes studied, asterisks denote significant differences between data of the pre-drug control and the GS967- or mexiletine-treated groups.

First, the effects of GS967 and mexiletine on I_NaL_ were studied under conventional voltage clamp conditions by applying 2 s duration depolarizations to −20 mV from the holding potential of −120 mV (Fig. 1). 1 µM GS967 significantly reduced the density of I_NaL_, measured at 50 ms after the beginning of the pulse (from −0.313 ± 0.05 to −0.062 ± 0.01 A/F, corresponding to an 80.4 ± 2.2% reduction in the 6 myocytes studied). For comparison, this parameter was also significantly decreased by 40 µM mexiletine from −0.385 ± 0.036 to −0.156 ± 0.014 A/F (reduction of 59.1 ± 1.8%, n = 12). Similar results were obtained when comparing the current integrals, i.e. the charge carried by the current with the exclusion of the initial 20 ms. As determined from the same experiments, the current integrals were significantly decreased from −69.1 ± 7.9 to −15.4 ± 3.9 mC/F (79.0 ± 3.1% inhibition) by GS967 and from −76.4 ± 7.6 to −26.7 ± 2.6 mC/F (63.3 ± 2.7% reduction) by mexiletine.

**Figure 2:**
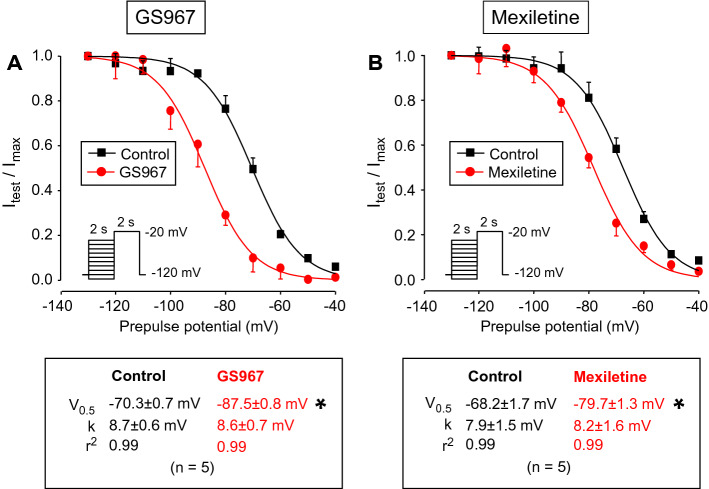
Effects of 1 µM GS967 (**A**) and 40 µM mexiletine (**B**) on the voltage dependence of steady-state inactivation of I_NaL_, determined using a conventional prepulse-protocol presented in the insets. 2 s long prepulses, ranging from –130 to −40 mV were followed by a test pulse clamped to −20 mV for 2 s. Each I_NaL_ amplitude (I_test_) was normalized to the peak current measured after the −130 mV prepulse (I_max_) and these I_test_/I_max_ ratios were plotted as a function of the corresponding prepulse-potential. Finally, data were fitted to a Boltzmann function and the results are given below the stead-state inactivation curves. Symbols and bars are mean ± SEM values, numbers in parentheses indicate the number of myocytes studied, asterisks denote significant differences between data of the pre-drug control and the GS967- or mexiletine-treated groups.

Since class I antiarrhythmics are known to modify inactivation kinetics of I_NaP_, the effects of 1 µM GS967 and 40 µM mexiletine on the voltage dependence of steady-state inactivation of I_NaL_ was also examined using a conventional prepulse protocol. The amplitude of I_NaL_ was determined at 50 ms after beginning of the test-pulse. Both GS967 and mexiletine caused a significant leftward shift in the steady-state inactivation curve (Fig. 2). The V_0.5_ value was shifted by −17.2 and −13.5 mV in the presence of GS967 and mexiletine, respectively. The slope factors of the curves were not influenced by GS967 or mexiletine.

### Effects of GS967 and mexiletine under action potential voltage clamp conditions

**Figure 3:**
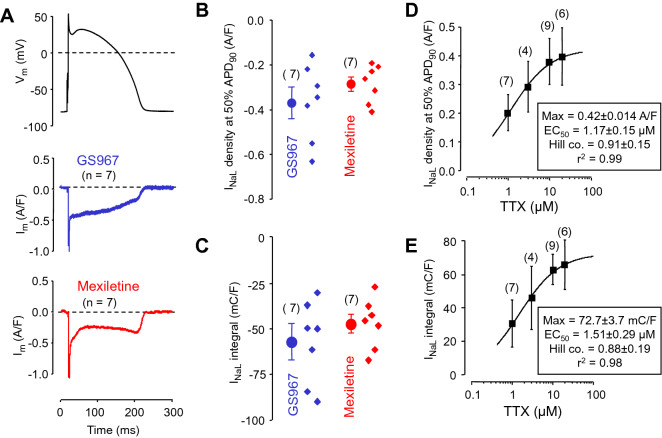
Effects of GS967 and mexiletine in isolated canine ventricular myocytes under action potential voltage clamp conditions. (**A**) Average membrane current profiles dissected by 1 µM GS967 and 40 µM mexiletine (in 7 myocytes in both cases), respectively, in the presence of 1 µM nisoldipine + 1 µM E4031 + 100 µM chromanol 293B. The command AP is shown above the current traces. (**B**) Current densities measured at 50% duration of APD_90_ of the command AP. (**C**) Current integrals from which the initial 20 ms period was excluded. (**D**,**E**) Concentration-dependent effects of TTX on the density and integral of I_NaL_. The solid line was obtained by fitting data to the Hill equation. For conventional reasons the polarity was ignored in these latter plots. Symbols and bars denote mean ± SEM values, small dots represent individual data, numbers in parentheses indicate the number of myocytes studied.

Based on the non-equilibrium gating theory of Clancy et al., gating of the Na^+^ channel is intimately influenced by the shape of the voltage protocol applied^33^. Therefore, the effects of GS967 and mexiletine on I_NaL_ were studied also under action potential voltage clamp conditions using canonic mid-myocardial APs as command signals. 1 µM GS967 and 40 µM mexiletine dissected inward I_NaL_ profiles having current densities (measured at 50% of APD_90_) of −0.37 ± 0.07 and −0.28 ± 0.03 A/F, and current integrals of −56.7 ± 9.1 and −46.6 ± 5.5 mC/F, respectively. These differences, however, were not significant statistically. Since the Ca^2+^ and K^+^ currents were previously blocked in these experiments, the recorded current profiles are considered as I_NaL_ inhibited by GS967 and mexiletine during a regular ventricular AP (Fig. 3A–C).

The total density and integral of I_NaL_ in canine myocytes were estimated using concentration–response curves obtained with TTX, which was applied as a reference compound. The maximum value of the Hill-fit gave the total magnitude of I_NaL_ (Fig. 3D,E). Comparing the respective current densities and integrals dissected by 1 µM GS967 and 40 µM mexiletine to these maximal values obtained with TTX, the inhibiting effects of GS967 and mexiletine could be expressed in a percentage form. Accordingly, GS967 and mexiletine blocked 88% and 67% of total I_NaL_ density, and 78% and 64% of total I_NaL_ integral, respectively. More importantly, the current profiles dissected by GS967 and mexiletine were different in shape. The GS967-sensitive current decreased monotonically during the AP, similarly to the TTX-sensitive current (not shown), while the mexiletine-sensitive current displayed a saddle-like profile. This difference is likely attributable to the threefold faster dissociation of GS967 than mexiletine from the Na^+^ channel (see later).

### Effects of GS967 and mexiletine on action potential upstroke

**Figure 4:**
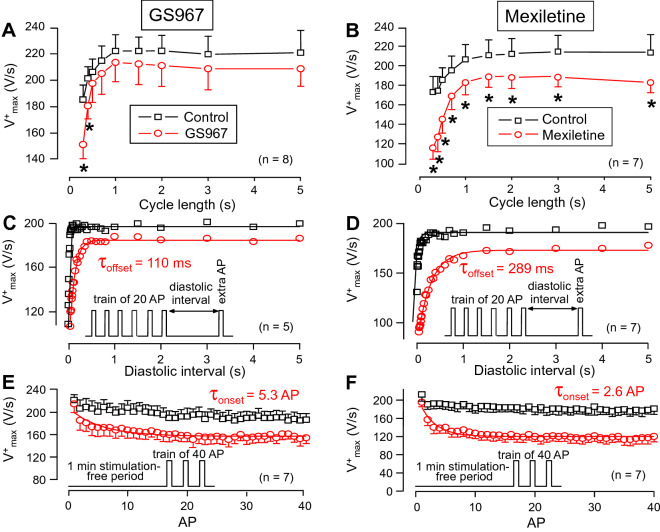
Effects of 1 µM GS967 (**A**,**C**,**E**) and 40 µM mexiletine (**B**,**D**,**F**) on the maximal rate of depolarization (V^+^_max_) measured in canine right ventricular trabeculae. (**A**,**B**) Cycle length-dependent effects on V^+^_max_ under steady-state conditions. (**C**,**D**) Determination of offset kinetics of GS967 and mexiletine as indicated by the time-dependent restitution of V^+^_max_. (**E**,**F**) Determination of the onset kinetics of the V^+^_max_-block at 2.5 Hz. The respective protocols are shown in the insets — for further details see Methods section. Solid lines were obtained by monoexponential fitting. Symbols and bars are mean ± SEM, numbers in parentheses indicate the number of preparations studied, asterisks denote significant differences between data of the pre-drug control and the GS967- or mexiletine-treated groups.

After determining the effects on I_NaL_, the actions of GS967 and mexiletine on I_NaP_ were studied and compared. Since direct measurement of cardiac I_NaP_ is difficult at 37 °C, the maximum velocity of depolarization during the action potential upstroke (V^+^_max_) was used as an approximate, although not linear, measure of I_NaP_^34–36^. Due to the more physiological situation (e.g. higher stability) in multicellular cardiac preparations, these experiments were performed in right ventricular papillary muscles using sharp microelectrodes. As demonstrated in Fig. 4A,B, 40 µM mexiletine significantly reduced V^+^_max_ in the entire frequency range applied under steady-state conditions, while this effect of GS967 was significant only at the shortest cycle lengths of 0.3 and 0.4 s. This difference can well be explained by the faster offset kinetics of GS967 (Fig. 4C,D). The time constant of recovery of V^+^_max_, determined following a constant 1 Hz stimulation was 110 ms for GS967, while almost three times longer, 289 ms for mexiletine. The onset kinetics of V^+^_max_ block was studied by application of a constant stimulation rate at 2.5 Hz and the initial 40 APs were recorded. The onset rate constant was 5.3 AP for 1 µM GS967 and 2.6 AP for 40 µM mexiletine (Fig. 4E,F).

### Effects of GS967 and mexiletine on APD

**Figure 5:**
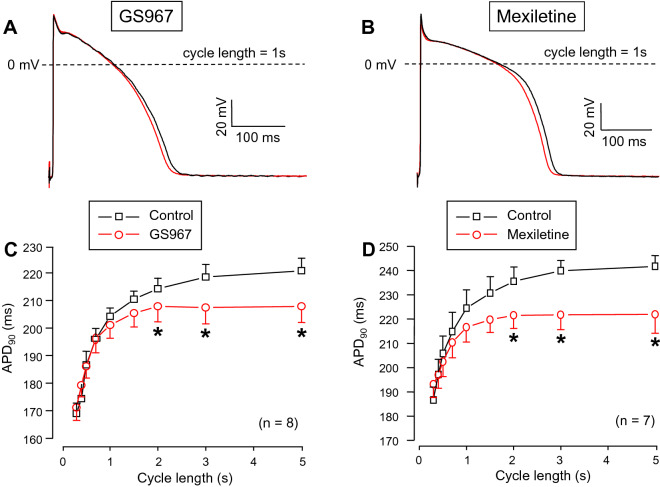
Cycle length-dependent **e**ffects of GS967 (**A**,**C**) and mexiletine (**B**,**D**) on AP duration measured at 90% of repolarization (APD_90_) in canine right ventricular trabeculae under steady-state conditions. During the experiments the cycle length was gradually changed between 0.3 and 5 s. (**A**,**B**) Representative superimposed analogue action potential pairs obtained at the constant pacing cycle length of 1 s before and after superfusion with 1 µM GS967 or 40 µM mexiletine. (**C**,**D**) Average rate-dependent APD values. Symbols and bars are mean ± SEM, numbers in parentheses indicate the number of preparations studied, asterisks denote significant differences between data of the pre-drug control and the GS967- or mexiletine-treated groups.

Although APD in right ventricular trabeculae was shortened by both GS967 and mexiletine in a reverse rate-dependent manner, this effect was statistically significant only at the longest constant cycle lengths above 1.5 s (Fig. 5).

### Effects of GS967 and mexiletine on beat-to-beat variability of APD

**Figure 6:**
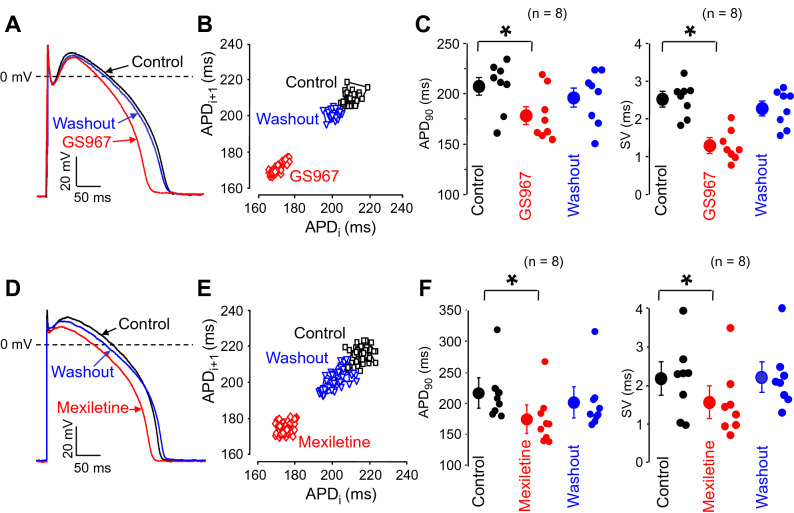
Reversible effects of GS967 (**A**–**C**) and mexiletine (**D**–**F**) on beat-to-beat variability of APD in isolated myocytes. Superimposed sets of action potentials (**A**,**D**) and Poincaré plots (**B**,**E**) obtained in control, following exposure to 1 µM GS967 or 40 µM mexiletine, and after washout of drugs. (**C**) and (**F**) show the average effects on beat-to-beat variability (SV) and APD. Symbols and bars are mean ± SEM, small dots represent individual data, numbers in parentheses indicate the number of myocytes studied, asterisks denote significant differences between data of the pre-drug control and the GS967- or mexiletine-treated groups.

Elevated beat-to-beat QT interval variability is a good predictor of ventricular arrhythmia in a wide variety of patients, while at a single cell level it translates into beat-to-beat variability of APD (also called short term variability, SV) — both parameters are considered as indicators of proarrhythmic risk^37–40^. In contrast to multicellular preparations, where the neighboring cells may effectively balance the individual differences in APD, SV is more pronounced in single cells, therefore the effects of GS967 and mexiletine were studied on SV in isolated ventricular cells, paced at a constant cycle length of 1 s. Under these conditions SV was significantly decreased by 1 µM GS967 and 40 µM mexiletine (reduction of 1.01 ± 0.19 and 0.63 ± 0.02 ms, respectively, corresponding to 42.1 ± 6.5% and 24.6 ± 12.8% decrease), while APD was moderately shortened by 32.5 ± 6.9 and 41.4 ± 4.1 ms, respectively, (all p < 0.05 and n = 8). All these effects were largely reversible upon washout (Fig. 6). Since APD itself is also known to affect the magnitude of SV, the term of relative SV (RSV) was introduced^31,41^. Accordingly, RSV = dSV / dAPD, i.e. the change in SV is normalized to that of APD. The value of RSV was significantly higher for GS967 than mexiletine (0.039 ± 0.007 *versus* 0.015 ± 0.007) predicting a better antiarrhythmic effectivity of GS967 than mexiletine.

## Discussion

We studied and compared the effects of GS967 (1 µM) to those of the class I/B antiarrhythmic drug mexiletine (40 µM) on I_NaL_ and I_NaP_ in canine ventricular myocardium by combining the conventional microelectrode, voltage clamp and action potential voltage clamp techniques. It was found that GS967, which is generally considered as a selective blocker of I_NaL_^16^, inhibits I_NaP_ as well, similarly to the class I/B antiarrhythmic drug mexiletine, but with higher potency. Based on the hypothetically selective I_NaL_ blocker nature of GS967, the drug was previously mentioned as a novel class VI antiarrhythmic agent^42^. However, without questioning the theoretical possibility of the concept for selective I_NaL_ inhibition, an alternative approach should also be considered, since the concept whether I_NaL_ can be blocked selectively is an interesting and still unresolved issue. There is evidence that Na^+^ current in cardiac tissues may also be conducted by Na^+^ channels other than the cardiac specific Na_v_1.5 channels, which were suggested to contribute to both I_NaP_ and I_NaL_. However, proper functional evidence for their role is still lacking while considerable interspecies differences were observed^43–51^. If these channels play a role in I_NaL_ but not in I_NaP_, their pharmacological inhibition may result in selective I_NaL_ blockade. However, GS967 has not been shown to inhibit these types of sodium channels but it was reported to inhibit cardiac type Na_v_1.5 channels^9,10^. Therefore‚ it is difficult to estimate the relative contribution of neuronal type Na^+^ channels to the GS967-sensitive current.

Na_v_1.5 channels have complex and multiple open and closed states^52^ with different drug binding properties governing active, inactive and resting channel states. Accordingly, drugs interacting with the binding sites of the channels depending on their actual open or closed channel states may produce variable effects on I_NaP_ and I_NaL_. For example, when a drug binds rapidly and with high affinity to open and inactivated channel states, and it dissociates rapidly from closed resting channel states, it would not inhibit I_NaP_ but I_NaL_. This would be due to complete drug dissociation from its binding site in the resting closed states unless frequency was very high or at least the cycle length would be shorter than its dissociation from the channel. Consequently, whether a drug inhibits I_NaP_ or I_NaL_ selectively or both of them, largely depends on the stimulation protocol but not on existing specific I_NaP_ or I_NaL_ binding sites. Present results and other recently published data^9,10^ are consistent with this suggestion and do not support a mechanism which is based on specific I_NaL_ inhibition that is distinctly different from class I/B antiarrhythmic actions described for drugs such as mexiletine^14,52^, lidocaine^9,10,14^, amiodarone^11^ and ranolazine^53^. This approach is also in line with the reported high (38-fold) selectivity of ranolazine on I_NaL_ over I_NaP_^2^, which is intermediate (13-fold) for amiodarone^3^ and much lower (only threefold) for flecainide^54^.

In our experiments, both GS967 and mexiletine significantly depressed V^+^_max_ at high stimulation rates. It was previously established that changes of V^+^_max_ and I_NaP_ are not linear and a relatively modest decrease of V^+^_max_ can represent robust depression of I_NaP_^35,36^. Accordingly, the 20–30% reduction of V^+^_max_ measured in the present study in papillary muscle preparations (at pacing cycle lengths of 0.3–0.4 s) following GS967 and mexiletine application can represent a similar degree of I_NaP_ depression as the measured 60–80% reduction of I_NaL_ obtained in ventricular myocytes. Therefore, in theory, neither GS967 nor mexiletine can be considered as “selective” I_NaL_ inhibitors. However, when therapy is concerned — assuming that decreased I_NaP_ is proarrhythmic, while reduced I_NaL_ is antiarrhythmic — GS967, which has about threefold faster offset kinetics, can be more beneficial than mexiletine since I_NaP_ would be affected in a lesser extent than I_NaL_ by GS967 at normal or moderately enhanced heart rates.

On the basis of present and other results it is clear that GS967 affects I_NaP_ in a strongly rate- and moderately species-dependent fashion. Similarly to our results, V^+^_max_ was reduced by 0.3 µM GS967 in murine myocytes^55^, while the same concentration of the drug failed to modify V^+^_max_ in canine Purkinje strands^56^. On the other hand, GS967 shortened APD in a variety of preparations, including rat^57,58^, murine^55^, rabbit^16,59^ and human^60^ ventricular cells within a wide concentration range (0.1–1 µM) — similarly to the present observations in isolated canine ventricular cells. In our multicellular preparations, however, a significant APD shortening effect appeared only at cycle lengths longer than 1.5 s. This can be explained by the well-known higher drug-sensitivity of single cells comparing to multicellular preparations. The reduction of I_NaL_ by GS967 may result the GS967-induced shortening of the elongated APD, reduction of the enhanced dispersion and short term variability of repolarization, changes often preceding torsade de pointes arrhythmias^38–40^.

Taken together the present results and the literature, it is likely that a compound having kinetic properties similar to GS967 would be a very promising new antiarrhythmic agent, since several in vitro^16–19,55–60^ and in vivo^57,61^ studies support the potent antiarrhythmic activity of GS967. Its kinetic properties are better than those of mexiletine, as shown in this study, and also than those of ranolazine^9^, agents known to suppress I_NaL_. GS967 had high brain penetration^62^, which can be utilized as a potential new antiepileptic compound^63^, and caused a profound use-dependent block on all sodium channel isoforms studied, making the compound prone for possible central nervous system side effects. It is unclear at present whether the effects of GS967 on the nervous system preclude the use of this drug as an antiarrhythmic therapeutic, however, a new agent exhibiting the same kinetic properties of GS967 without CNS side effects would represent a promising candidate for future development.

In summary, GS967 —similarly to mexiletine— inhibited both the peak and late components of Na^+^ current and decreased beat-to-beat variability of APD. Based on its kinetic properties, GS967 should be classified as a new potent class I/B antiarrhythmic agent. The results of the present study also suggest that investigations of “selective” I_NaL_ inhibitors should be carried out through a wide range of stimulation rates since the effect of drugs like GS967 or mexiletine, that possess fast offset kinetics of I_NaP_ inhibition, can be misinterpreted.
